# 406. Effectiveness of the JN.1-adapted BNT162b2 COVID-19 Vaccine in High-Risk Groups against Hospitalization in Europe: A Test-Negative Case-Control using the id.DRIVE Platform

**DOI:** 10.1093/ofid/ofaf695.013

**Published:** 2026-01-11

**Authors:** Hannah R Volkman, Leonie de Munter, Thao M P Tran, Cátia Marques, Elisabeth J Mesfun-Kap, Laura Choi, Hollie Dunford, Srinivas Valluri, Juan V Hernandez-Villena, Jingyan Yang, Sultan Abduljawad, Irma Casas, Elisa Martró, Beate Grüner, Ainara Mira-Iglesias, Carlos M Oñoro-López, Oleguer Parés-Badell, Alejandro Orrico-Sánchez, Susana Otero-Romero, Antoni Torres, Federico Martinon-Torres, Jennifer L Nguyen, Kaatje Bollaerts

**Affiliations:** Pfizer, New York, New York; P95 Epidemiology and Pharmacovigilance, Leuven, Brabant Wallon, Belgium; P95 Clinical and Epidemiology Services, Leuven, Vlaams-Brabant, Belgium; Pfizer Inc., Lisbon, Lisboa, Portugal; P95 Clinical and Epidemiology Services, Leuven, Vlaams-Brabant, Belgium; Pfizer Inc., Lisbon, Lisboa, Portugal; P95 Clinical and Epidemiology Services, Leuven, Vlaams-Brabant, Belgium; Pfizer Inc, New York, New York; P95 Clinical and Epidemiology Services, Leuven, Vlaams-Brabant, Belgium; Pfizer and Columbia University Institute for Social and Economic Research and Policy, New York, New York; BioNTech UK Ltd., London, England, United Kingdom; Hospital Universitari Germans Trias i Pujol, Badalona, Catalonia, Spain; Hospital Universitari Germans Trias I Pujol, Badalona, Catalonia, Spain; University Hospital Ulm, Ulm, Baden-Wurttemberg, Germany; Fisabio Public-Health, Valencia, Comunidad Valenciana, Spain; Hospital Universitario La Paz, Madrid, Madrid, Spain; Hospital Universitari Vall d'Hebron, Barcelona, Catalonia, Spain; FISABIO-Public Health, Valencia, Comunidad Valenciana, Spain; Hospital Universitario Vall d’Hebrón, Barcelona, Catalonia, Spain; Hospital Clinic, University of Barcelona, IDIBAPS, CIBERES, Barcelona, Catalonia, Spain; Hospital Clínico Universitario de Santiago, Santiago de Compostela, Spain, Santiago de Compostela, Galicia, Spain; Pfizer Inc., Lisbon, Lisboa, Portugal; P95 Epidemiology and Pharmacovigilance, Leuven, Brabant Wallon, Belgium

## Abstract

**Background:**

There is limited information on COVID-19 vaccine effectiveness (VE) among specific groups at high risk for severe COVID-19, including older adults and patients with chronic conditions. We report on the VE of the JN.1-adapted BNT162b2 vaccine in various high-risk groups.

**Table 1.**
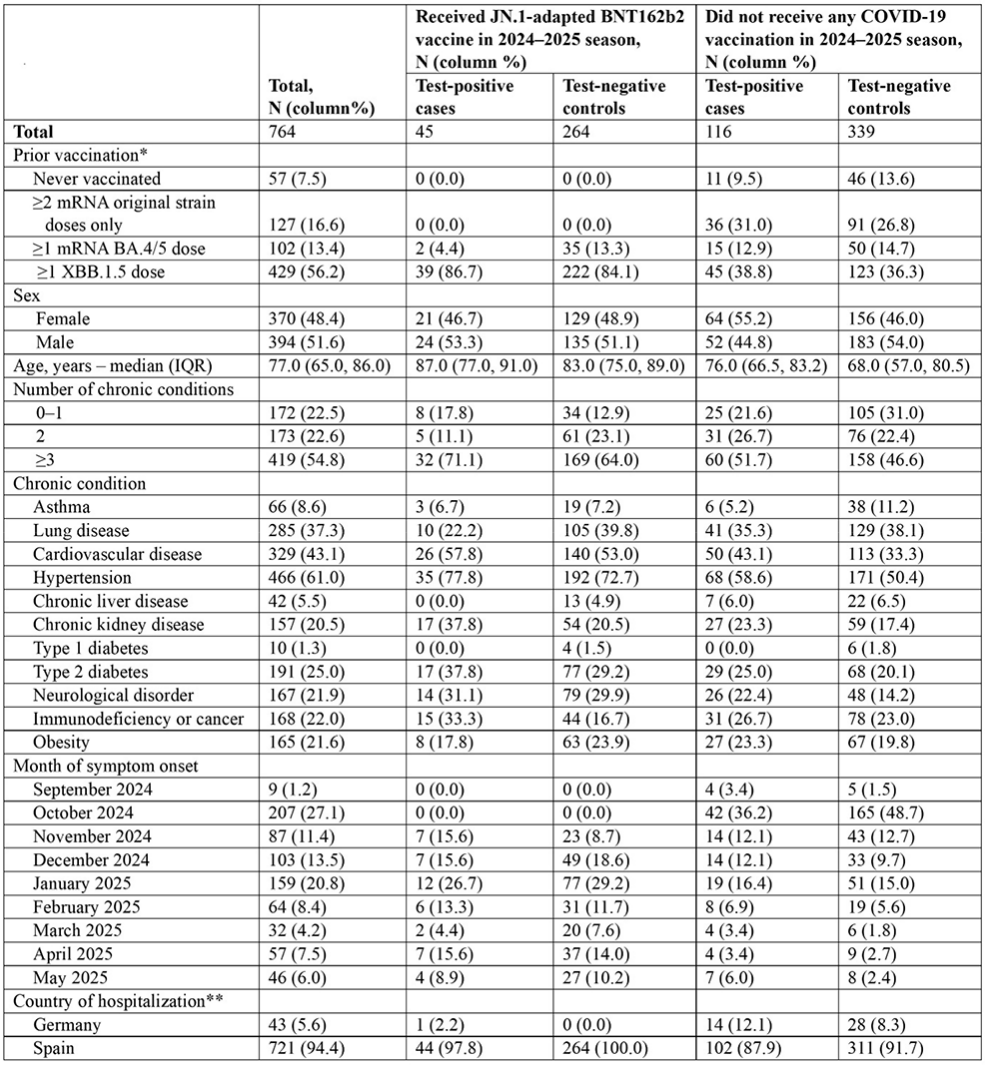
Characteristics of study patients hospitalized with severe acute respiratory infection according to JN.1-adapted BNT162b2 vaccine receipt and SARS-CoV-2 status, in Europe, September 2024–May 2025 *Does not include vaccination regimens with vaccines not mentioned here (n=49) ** Study sites included 1 hospital located in Germany and 10 hospitals located in Spain.

**Figure 1.**
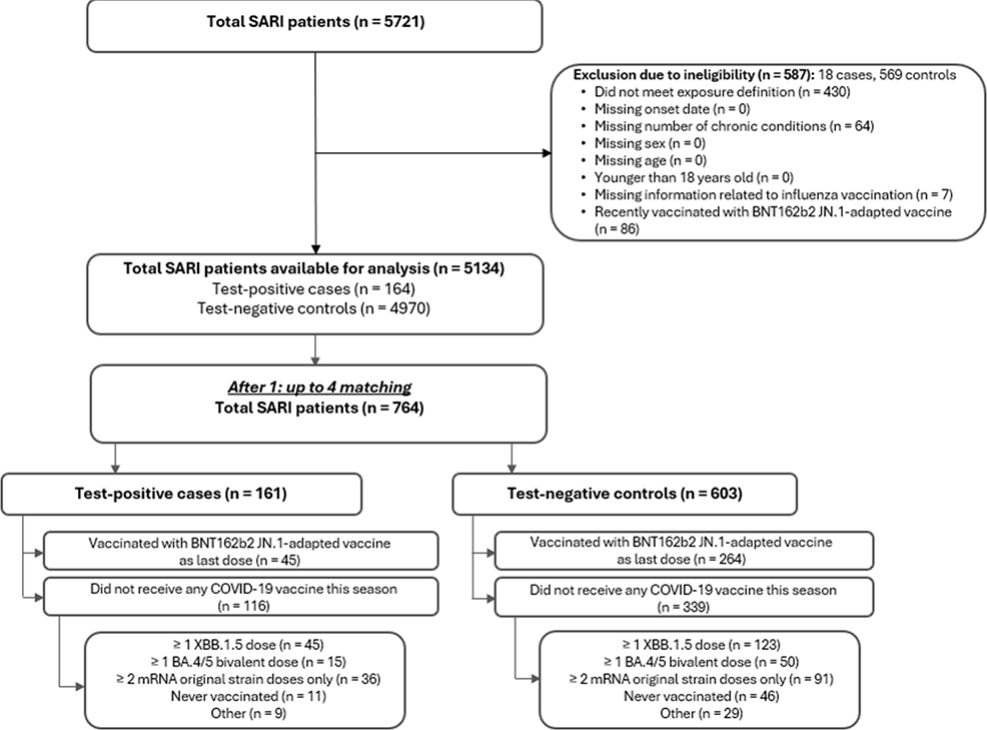
Flow diagram of the study population in id.DRIVE, September 2024–May 2025 SARI: severe acute respiratory infection

**Methods:**

We conducted a test-negative case-control study in the id.DRIVE platform (EUPAS42328) of adults ≥18 years hospitalized with a severe acute respiratory infection in sites across Germany and Spain from 3 September 2024–31 May 2025. Vaccination status was determined using registries or medical documentation. Exposed patients received ≥1 dose of the JN.1-adapted BNT162b2 vaccine, while unexposed patients did not receive any COVID-19 vaccine in the 2024–2025 season. SARS-CoV-2 status was confirmed by PCR. Multivariable analyses were adjusted for symptom onset date, age, sex, number of chronic conditions, and influenza vaccination status. Cases were matched with up to 4 controls by site and symptom onset date (two-week interval). Adjusted VE was calculated as (1− odds ratio) × 100%.

**Figure 2.**
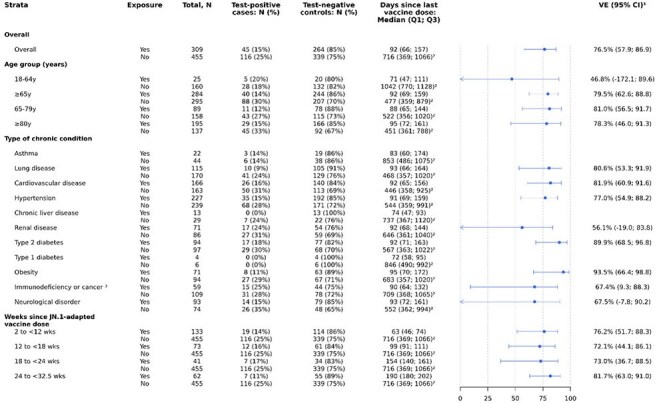
Vaccine effectiveness against COVID-19 hospitalization in patients with a severe acute respiratory infection who received at least one dose of JN.1-adapted BNT162b2 vaccine compared with patients who did not receive any dose of a COVID-19 vaccine in the 2024–2025 season CI confidence interval; N: number; Q1/Q3: quartile 1/3; VE: Vaccine effectiveness; wks: weeks; y: years. ¹Vaccine effectiveness estimates are adjusted for date of symptom onset, age, sex, number of chronic conditions, and receipt of influenza vaccine in the 12 months prior to current hospital admission. ²’Never vaccinated’ study participants were excluded when calculating the median (IQR) of time since last vaccine dose in the unexposed group (patients who did not receive any dose of a COVID-19 vaccine in the 2024–2025 season) ³The categories ‘Cancer’ and ‘Immunodeficiency’ among chronic conditions are counted as separate types of chronic conditions in all adjusted VE estimates. Here, the VE estimate among participants having ‘Immunodeficiency or cancer’ is provided as a composite.

**Results:**

A total of 764 patients were included in the analysis, including 161 cases and 603 controls, of whom 309 (40.4%) received a JN.1-adapted BNT162b2 vaccine. Overall, VE against hospitalization was 76.5% (95% CI: 57.9; 86.9) at a median 92 days (IQR 66; 157) since vaccination with sustained protection through 24–32 weeks since dose. VE was similar across age groups; VE was 81.0% (95% CI: 56.5; 91.7) among adults 65–79 years and 78.3% (95% CI: 46.0; 91.3) among adults ≥80 years. BNT162b2 was effective among adults with specific chronic conditions, including lung disease, cardiovascular disease, hypertension, type 2 diabetes, and obesity, while estimates lacked precision for asthma, chronic liver disease, renal disease, type 1 diabetes, and neurological disorder. VE was 67.4% (95% CI: 9.3; 88.3) among patients with immunodeficiency or cancer.

**Conclusion:**

The JN.1-adapted BNT162b2 vaccine was effective against hospitalization during the 2024–2025 season in a range of high-risk groups and provided durable protection lasting through 6 months. These findings support the clinical benefit of BNT162b2 among groups at high-risk for severe disease and emphasize the continued importance of COVID-19 vaccination in high-risk patients.

**Disclosures:**

All Authors: No reported disclosures

